# Light-induced shift current vortex crystals in moiré heterobilayers

**DOI:** 10.1073/pnas.2314775120

**Published:** 2023-12-12

**Authors:** Chen Hu, Mit H. Naik, Yang-Hao Chan, Jiawei Ruan, Steven G. Louie

**Affiliations:** ^a^Department of Physics, University of California at Berkeley, Berkeley, CA 94720; ^b^Materials Sciences Division, Lawrence Berkeley National Laboratory, Berkeley, CA 94720; ^c^Institute of Atomic and Molecular Sciences, Academia Sinica, and Physics Division, National Center for Theoretical Sciences, Taipei 10617, Taiwan

**Keywords:** shift current, moiré heterobilayers, vortex crystals, time-dependent GW

## Abstract

Transition metal dichalcogenide (TMD) moiré superlattices provide an emerging platform to explore various light-induced phenomena. Recently, the discoveries of novel moiré excitons have attracted great interest. The nonlinear optical responses of these systems are however still underexplored. Here, we report investigation of light-induced shift currents (a second-order response generating DC current from optical illumination) in the WSe_2_/WS_2_ moiré superlattice. We identify a striking phenomenon of the formation of shift current vortex crystals—i.e., two-dimensional periodic arrays of moiré-scale current vortices and associated magnetic fields with remarkable intensity under laboratory laser setup. Furthermore, we demonstrate high optical tunability of these current vortices—their location, shape, chirality, and magnitude can be tuned by the frequency, polarization, and intensity of the incident light. Electron-hole interactions (excitonic effects) are found to play a crucial role in the generation and nature of the shift current intensity and distribution. Our findings provide a promising all-optical control route to manipulate nanoscale shift current density distributions and magnetic field patterns, as well as shed light on nonlinear optical responses in moiré quantum matter and their possible applications.

## Significance

Employing light to drive different phenomena or to induce and tune exotic phases of matter lies at the heart of modern condensed matter science and technologies such as optoelectronics. Shift current, a second-order optical response in noncentrosymmetric materials, is a notable DC photocurrent (generated without a p-n junction) having prominent features like low dissipation and robustness against scattering. We investigate shift current generation and their microscopic real-space density distributions in WSe_2_/WS_2_ moiré heterobilayers. We identify an moiré quantum matter—light-induced shift current vortex crystals with associated magnetism—as well as an all-optical control route for its manipulation. These findings offer insight into the photophysics of van der Waals moiré systems and open rich opportunities for their applications.

The bulk photovoltaic effect (BPVE) is a general term that refers to the DC electric current generation in noncentrosymmetric materials under illumination of optical light (1–4). Unlike conventional photovoltaic devices, BPVE does not require a p-n junction or external bias to separate the photoexcited electrons and holes for a DC current, providing a fundamentally new route for high-efficiency photovoltaics (5–13). Shift current, which is an intrinsic mechanism for BPVE, is a second-order optical response and could conceptually be interpreted as the “shift” of the intracell coordinates of the excited electrons (14–22). In contrast to drift current, light-induced shift current is of a pure quantum nature which originates from the spatial evolution of electronic wavepackets from photoexcitations. Previous studies (1–22) have shown that shift current has deep connections with the topological properties of the electronic states and exhibits various useful features—such as low dissipation and robustness against scattering. In low-dimensional materials, a recent study moreover showed that electron-hole interaction (excitonic) effects play a key role in enhancing and modifying shift currents (22).

Moiré superlattices created by vertically stacking of van der Waals two-dimensional (2D) materials with small lattice mismatches and/or layers with different orientations offer a powerful platform for exploring novel physical phenomena, including topological phases (23, 24), ferromagnetism (25–28), unconventional superconductors (29), and correlated insulators (30, 31). At the same time, due to strong light-matter interactions, transition metal dichalcogenide (TMD) moiré superlattices have opened up opportunities for a variety of light-induced excitonic phenomena (32–39). In particular, novel moiré excitons with distinct spatial characters (32–34) have been discovered. Moiré modulation effects on photoexcitation physics lie in the heart of both the fundamental understanding of moiré exciton states and the possible applications of these systems in optoelectronics. Despite impressive progress on the linear optical response of TMD moiré superlattices (32–34) (e.g., absorbance), so far, their nonlinear optical responses remain underexplored both theoretically and experimentally.

Here, we report ab initio investigations of light-induced shift currents and their microscopic real-space distributions in rotationally aligned WSe_2_/WS_2_ moiré superlattices. To capture accurately many-body excitonic effects from first principles, we employ a time-dependent adiabatic *GW* (TD-a*GW*) approach with real-time propagation of the density matrix in the presence of the external light field (22). Our findings show that electron-hole interaction effects play a crucial role in the character and magnitude of the shift current. The current density arising from illumination of light with different frequency (corresponding to transitions to different moiré exciton resonances) and polarization exhibit distinct real-space distributions. Importantly, we identify a striking phenomenon of the formation of 2D periodic arrays of shift current vortices and their induced magnetic field nanopatterns. Under illumination frequency corresponding to transitions to the intralayer charge-transfer excitons (32), current vortices of opposite circulation chirality are created within each moiré unit cell forming a well-defined vortex crystal. Linearly and circularly polarized light create distinct types of vortex crystals, which exhibit antiferromagnetism and ferrimagnetism, respectively. We further demonstrate that the frequency, polarization, and intensity of the incident light can efficiently tune the location, shape, chirality, and magnitude of these photoinduced current vortices, suggesting a promising all-optical control to photocurrent density distribution and associated magnetism in TMD moiré superlattices.

**Fig. 1.**
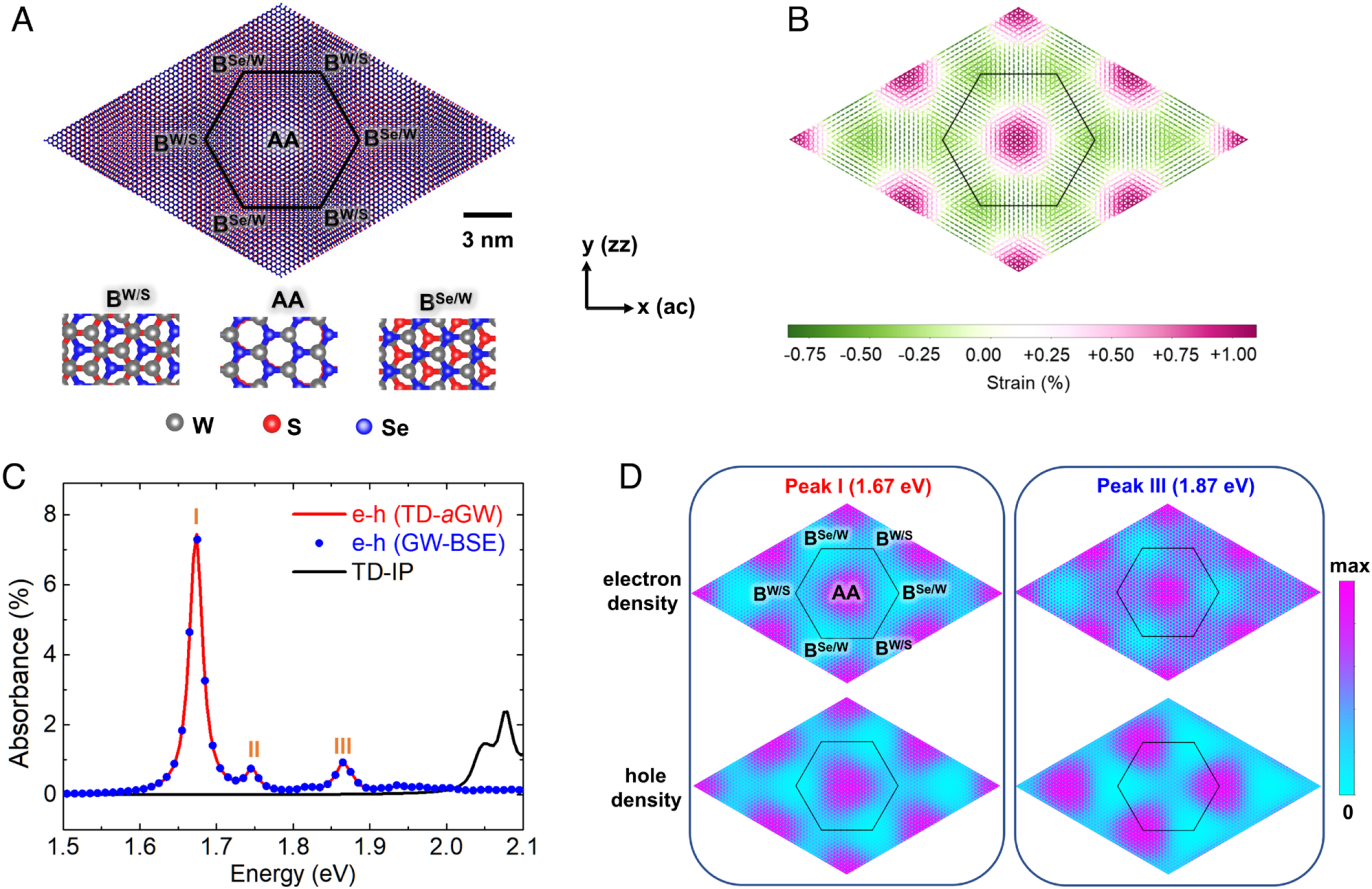
Moiré excitons of the WSe_2_/WS_2_ heterobilayer. (*A*) Atomic structure of rotationally aligned WSe_2_/WS_2_ moiré superlattice. The black outline denotes the hexagonal moiré supercell with lattice constant of 8.3 nm. The positions of three high-symmetry local stackings (AA, B^Se/W^, B^W/S^) are labeled, and the *X* axis and *Y* axis are aligned to armchair (ac) and zigzag (zz) directions, respectively. AA–W (Se) atoms of WSe_2_ are on the top of W(S) atoms of WS_2_, B^Se/W^–W (Se) atoms of WSe_2_ are on the hollow sites (top of W atoms) of WS_2_, and B^W/S^–Se (W) atoms of WSe_2_ are on the hollow sites (top of S atoms) of WS_2_. (*B*) Real-space strain distribution of the WSe_2_ layer which originate from the moiré structural reconstruction. (*C*) Computed absorbance of the WSe_2_/WS_2_ moiré superlattice: with electron-hole interactions (red solid line and blue dots from TD-a*GW* and *GW*-BSE approaches, respectively) and without electron-hole interactions (IP, black solid line). The positions of three moiré exciton resonance peaks (I, II, and III) are marked. A typical experimental spectral broadening (32) of 10 meV is used. (*D*) Real-space distributions of the electron densities $\rho_{e}\left(r_{e}\right)$ and hole densities $\rho_{h}\left(r_{h}\right)$ of exciton states forming peaks I and III, respectively, which are defined as $\rho_{e}\left(r_{e}\right)=\int {\left|\chi_{S}\left(r_{e},r_{h}\right)\right|}^{2}dr_{h}$ and $\rho_{h}\left(r_{h}\right)=\int {\left|\chi_{S}\left(r_{e},r_{h}\right)\right|}^{2}dr_{e}$ where $\chi_{S}$ is the wavefunctions of exciton with quantum index S and electron and hole coordinates $r_{e}$ and $r_{h}$. The integrals are taken over the whole crystal.

For rotationally aligned WSe_2_/WS_2_ bilayer, the lattice constant mismatch between the two layers is about 4%, giving rise to a hexagonal moiré supercell with periodicity about 8.3 nm (Fig. 1*A*). To minimize the total energy, the superlattice reconstructs to increase the areas of the lower energy Bernal (B^Se/W^ and B^W/S^) stacking regions while decrease the higher energy areas (AA stacking regions) (32, 40, 41). This leads to a remarkable moiré structural reconstruction with strain as shown in Fig. 1*B*: local compression in the Bernal stacking regions and local expansion in the AA regions in the WSe_2_ layer. Previous experimental and theoretical studies have revealed that this system has a type II band alignment and its low-energy optical properties for normal incident light are dominated by the WSe_2_ intralayer excitons because the interlayer excitons have very small oscillator strengths and the WS_2_ intralayer exciton excitations (with high energies) are well separated from the WSe_2_ resonances (32–34). The large-scale inhomogeneous strain field of the WSe_2_ layer (Fig. 1*B*) strongly modifies its band structure (leading to flat bands) and modulates the wavefunction of the quasiparticle moiré states in real space (32), resulting in the formation of novel moiré excitons.

In contrast to a single low-energy peak (A-exciton) of the pristine WSe_2_ monolayer (42, 43) in the energy range shown in Fig. 1*C*, there are three moiré excitation peaks in the computed absorbance spectra of the moiré superlattice which match well with recent optical measuments (32–34). We note that the absorbance can not be captured even qualitatively by the independent particle approximation (IP, black line). Also, the identical absorbances obtained by our TD-a*GW* approach (red line) and the standard *GW* plus Bethe–Salpeter equation (*GW*-BSE) method (blue dots) validate the accuracy of the excitonic effects included in the TD-a*GW* calculations for large-scale TMD moiré systems. The formalism and computational details on the *GW* (44), *GW*-BSE (45, 46), and TD-a*GW* (22) methods can be found in the *Methods*. As shown in ref. 32 and Fig. 1, the intralayer moiré excitons (peaks I, II, and III) of this system possess distinct microscopic character (Fig. 1*D*): The moiré exciton of peak I (lower energy, 1.67 eV) has the Wannier-type character where the correlated electron and hole densities coincide in space and are located around the AA stacking region, while the moiré exciton of peak III (higher energy, 1.87 eV) exhibits an intralayer charge-transfer character where the correlated electron and hole densities are spatially separated and are located at the AA and B^Se/W^ stacking regions, respectively. Peak II (middle energy, 1.75 eV), on the other hand, has a mixed character.

In the following, we present our results on the quantity of central interest of this work—the real-space distributions of light-induced microscopic shift current density. The local current density $J\left(r,t\right)$ in a time-dependent driving field for a quantum system is generally obtained from the expectation value of the current density operator $\hat{J}\left(r\right)=\frac{e}{2}[\left|r\rangle \langle r\left|\hat{v}+{\hat{v}}^{†}\right|r\rangle \langle r\right|]$, where e is the charge of the electron, and $\hat{v}$ is the velocity operator. This may be achieved using the density matrix ρ(t) through $J\left(r,t\right)=tr\left[\rho \left(t\right)\hat{J}\left(r\right)\right]$. We compute the interacting ρ(t) using the ab initio TD-a*GW* approach (see *Methods*). In the Bloch-state single-particle orbital basis, the optically induced local current density is then given by

[1] $$J\left(r,t\right)=\frac{e}{2N_{k}}\sum_{nml,k}\rho_{nm,k}\left(t\right)[v_{ln,k}\psi_{m,k}^{*}\left(r\right)\psi_{l,k}\left(r\right)+v_{ml,k}\psi_{l,k}^{*}\left(r\right)\psi_{n,k}\left(r\right)],$$

where $\psi_{n,k}\left(r\right)$ is a Bloch state orbital with band index *n* and wavevector ***k,*** and $N_{k}$ is the number of k points in the Brillouin zone sampled. Using Fourier transformation, responses at different frequencies can be computed through $J\left(r,t\right)=\Sigma_{\omega }J\left(r,\omega \right)e^{i\omega t}$ and the shift current density at position r is given by the DC component $J^{\mathrm{DC}}\left(r\right)=J\left(r,\omega =0\right)$. In some previous studies, the terms in Eq. **1** involving the diagonal (n=m) and off-diagonal (n≠m) elements of the density matrix were separated and treated differently, and given different terminologies (3, 4). Such separation for the density matrix of an interacting many-particle system is conceptually unnecessary and basis dependent. In our calculations, the full density matrix is directly used to compute the complete real-space density distribution of the DC photocurrent to second order in the optical field (which we refer to as the shift current). In experiments of 2D systems, the 2D current density is a well-defined measurable quantity which describes the charge flow in the layer per unit length. Therefore, we integrate the computed 3D current density over the length of the supercell in the normal direction (z) in our simulation to obtain a 2D current density in the x–y plane. Throughout the rest of the paper, for the sake of simplicity, we will refer to the 2D DC photocurrent (shift current) density distribution as J(r) with $r=\left(x,y\right)$.

**Fig. 2.**
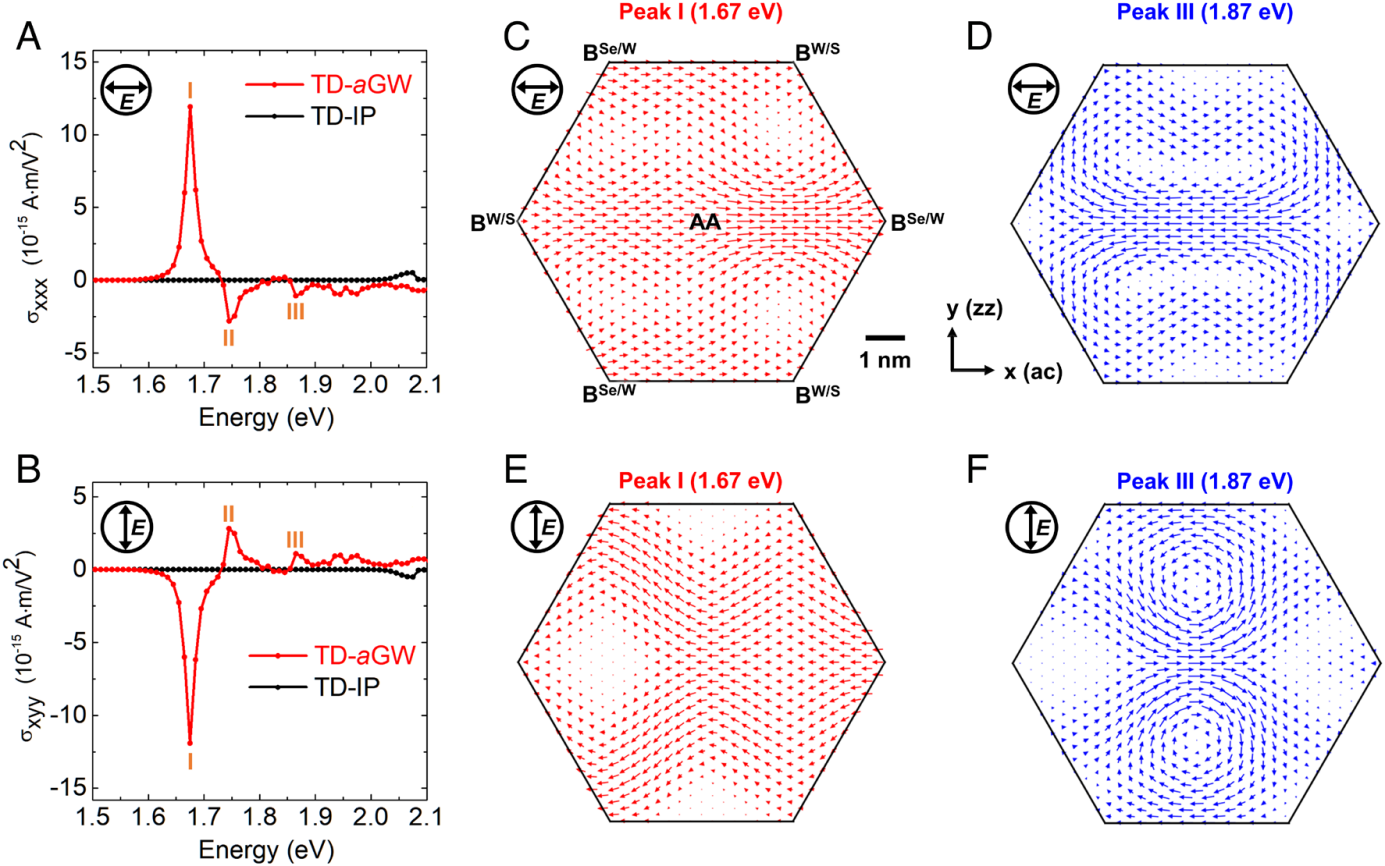
Shift currents of the WSe_2_/WS_2_ moiré superlattice. (*A* and *B*) Spectra of macroscopic shift current conductivity tensor components $\sigma_{\mathrm{xxx}}$ and $\sigma_{\mathrm{xyy}}$ that give rise to the *x* component of the macroscopic shift current density (${\bar{J}}_{x}$) with linear-polarized light field (**E**) along the *x*- and *y*-direction, respectively, as defined in Eq. **2**. Electron-hole interactions are included in the TD-a*GW* (red line) and excluded in the TD-IP (black line) calculations. (*C*–*F*) Microscopic shift current density $J\left(r\right)$ corresponding to incident light frequency at resonance with exciton peaks I and III with linearly polarization along the *x* direction (*C* and *D*) and *y* direction (*E* and *F*). The direction and amplitude of J(r) are represented by the orientation and length of the arrows.

In the literature, to our knowledge, only the shift current density averaged over the unit cell $\overset{¯}{J}=\frac{1}{\Omega }\int J\left(r\right)dr$ was studied for crystals, where Ω is the unit cell volume (or area in 2D). It is traditionally expressed, from integrating Eq. **1**, in the form (22):

[2] $${\bar{J}}_{a}=2\sigma_{\mathrm{abc}}\left(0;\omega ,-\omega \right)E_{b}\left(\omega \right)E_{c}\left(-\omega \right),$$

where E is the electric field of the incident light (assumed to be uniform over the sample) and a,b,c are Cartesian components. We shall call $\bar{J}$ the macroscopic current density. However, as shown below, it gives limited information compared to $J\left(r\right)$. The different components of the second-order macroscopic conductivity tensors ($\sigma_{\mathrm{abc}}$) are intrinsically connected by the global crystal symmetry of the system (47). Fig. 2 *A* and *B* show the spectra of $\sigma_{\mathrm{xxx}}$ and $\sigma_{\mathrm{xyy}}$ with Cartesian coordinates as defined in Fig. 1*A*, which hold the relation: $\sigma_{\mathrm{xxx}}=-\sigma_{\mathrm{xyy}}$, governed by the C_3v_ symmetry of the rotationally aligned WSe_2_/WS_2_ moiré supercell (47) (i.e., threefold axis with mirror symmetry planes along armchair directions). In general, $\sigma_{\mathrm{abc}}$ as a tensor in 2D has eight components; however, for our system with C_3v_ symmetry, $\sigma_{\mathrm{xxx}}=-\sigma_{\mathrm{xyy}}=-\sigma_{\mathrm{yyx}}=-\sigma_{\mathrm{yxy}}$, with all other components equal to zero (47). With inclusion of electron-hole interaction effects (red line) in the calculation, one can explicitly identify three prominent peaks (I, II, and III) in the computed shift current conductivity spectra (Fig. 2 *A* and *B*), corresponding to the three moiré exciton peaks in the absorbance (Fig. 1*C*). On the other hand, the results computed within the independent particle approximation (black line) are dramatically different and fail to capture these dominant moiré exciton features even qualitatively.

We find that the real-space distributions of the microscopic shift current density $J\left(r\right)$ exhibit significantly more complex characters and richer physics that are hidden from the macroscopic current density analysis. They are shown in Fig. 2 *C*–*F* for different linearly polarized light at frequencies corresponding to transitions to excitons I and III. First, as opposed to having a vanishing *y* component for the cell-averaged macroscopic current density $\bar{J}$ (i.e., with $\sigma_{\mathrm{yxx}}$ = $\sigma_{\mathrm{yyy}}=0$), the current density $J\left(r\right)$ has remarkably large *y* component at different positions ***r*** within the moiré cell, yielding large local current density along the *y* direction. Second, when changing the light linearly polarized from along the *x* direction to the *y* direction (or vice versa), although the macroscopic photocurrent conductivity only has a trivial sign-flipping ($\sigma_{\mathrm{xxx}}=-\sigma_{\mathrm{xyy}}$), the local current density $J\left(r\right)$ flows in prominently different pathways (comparing Fig. 2*C* with Fig. 2*E* or Fig. 2*D* with Fig. 2*F*). Third, with light polarized along the *y* direction and an intensity of $1.0\times 10^{10}$ W/m^2^ exciting exciton peaks I (1.67 eV) and III (1.87 eV), the cell-averaged macroscopic current density $\bar{J}$ is 0.09 A/m and 0.007 A/m, respectively—showing an order-of-magnitude difference in the current generated by exciting the two excitons. However, their maximum local current densities turn out to be very close: The computed maximum $J\left(r\right)$ for exciton I (Fig. 2*E*) and exciton III (Fig. 2*F*) are 0.16 A/m and 0.13 A/m, respectively. This reveals that the local shift current response for exciton III is as strong as that for exciton I. Exciton III’s much smaller cell-averaged value ($\bar{J}$) originates from the formation of current vortex structures as seen in Fig. 2 *D* and *F*.

**Fig. 3.**
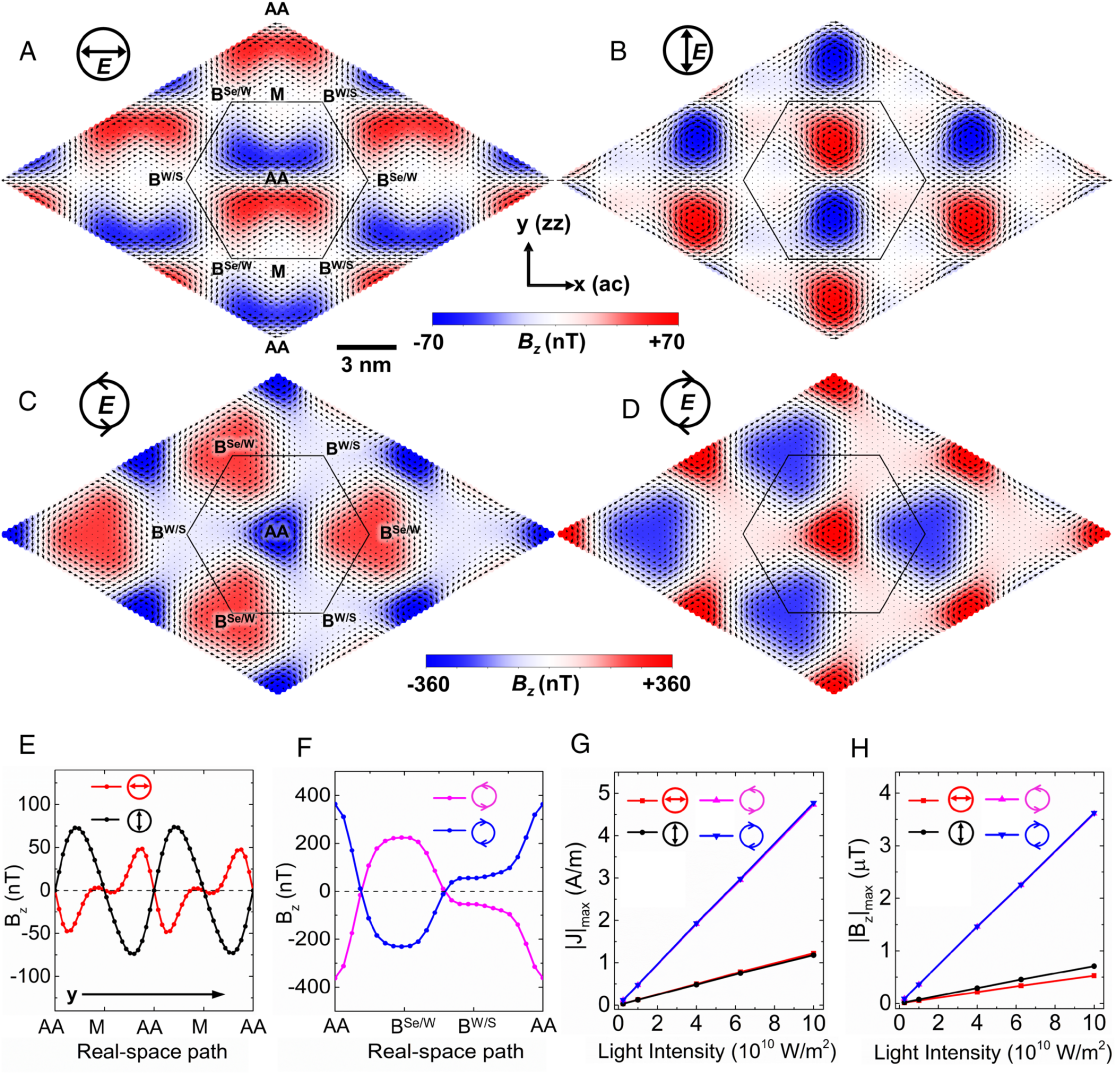
Moiré shift current vortices and induced magnetic field from exciton peak III. (*A*–*D*) Real-space plot of the microscopic shift current density J(r) (black arrows) and the current-induced out-of-plane magnetic field $B_{z}\left(r\right)$ evaluated on the top of the Se atomic plane (color coding), produced by light field ***E*** with frequency of the exciton peak III (1.87 eV), with linear light polarization along the *x* direction (*A*) and *y* direction (*B*), and circular light polarization of left-handed helicity (*C*) and right-handed helicity (*D*). The upper (*Lower*) color-scale bar is for upper (*Lower*) two panels. (*E*) Induced magnetic field along the real-space path (along *y*): AA-M-AA (see marks in *A*) with different linear polarizations. (*F*) Induced magnetic field along the real-space path: AA-B^Se/W^-B^W/S^-AA (see marks in *C*) with different circular polarizations. In *A*–*F*, results are for a light intensity of $1.0\times 10^{10}$ W/m^2^. (*G* and *H*) Maximum microscopic photocurrent density (*G*) and induced magnetic field (*H*) as a function of incident light intensity for different linear and circular polarizations.

Our ab initio TD-a*GW* results predict that with light frequency in resonance with moiré exciton peak III, two current vortices are generated in each moiré supercell (in the WSe_2_ layer). For in-plane linear polarizations, the two vortices have the same magnitude but opposite circulation chirality, residing on two sides (*Upper* and *Lower* in Fig. 2 *D* and *F*) separated by the mirror line (y=0). With light polarization direction switching from along the *x* direction (Fig. 2*D*) to along the *y* direction (Fig. 2*F*), the chirality of the current vortices flips: The vortex located at the upper (lower) side changes from clockwise (counterclockwise) to counterclockwise (clockwise), revealing a high optical tunability of the shift current flow. Additionally, it is found that changing the polarization direction of the linearly polarized light also significantly modulates the location and shape of the current vortices. More details of light-polarization-direction dependence of the shift current vortices are given in *SI Appendix*. For in-plane circular polarizations, the two vortices in the moiré supercell are still of opposite chirality but now have very different amplitude (Fig. 3 *C* and *D*), leading to a net chiral current within a moiré cell, which changes sign upon changing from left to right circular polarization (or vice versa). This phenomenon of having vortex pairs of opposite chirality can be understood generally from differential topology since the microscopic current density generated by uniform light illumination in a 2D periodic crystal can be mapped to a tangential vector field on a compact 2D surface. The Poincaré–Hopf theorem stipulates that the sum of the Poincaré indices at critical points (the winding numbers of vortices in our case) is equal to zero for a 2D torus. A moderate-size moiré superlattice that supports a charge-transfer exciton with appreciable oscillator strength would be optimal for observing shift current vortices. This is because very small-size superlattices might not host in-plane charge-transfer excitons (34), while excessively large-size ones may lead to negligible oscillator strength of such excitons.

The formation of a periodic array of photocurrent vortex pairs induced by optical transitions results in a distinctive moiré-scale vortex crystal with fascinating magnetic properties. As discussed above, for in-plane linearly polarized light with frequency exciting exciton III, two vortices reside on two sides of the moiré cell (*Upper* and *Lower* in Fig. 2 *D* and *F*) separated by the y=0 mirror line. They produce a 2D array of nanoscale antiparallel magnetic fields in the moiré superlattice, as shown in Fig. 3 *A* and *B*. Since the two current vortices have the same shape, magnitude but opposite circulation chirality, they yield a vanishing net magnetic flux and well-defined antiferromagnetism on the moiré supercell scale. On the other hand, upon illumination by circularly polarized light (Fig. 3 *C* and *D*), two distinct sets of current vortices are generated in the moiré superlattices residing on the AA and B^Se/W^ stacking regions, respectively. These two vortices possess very different magnitude, shape, size, and circulation chirality, giving rise to a net finite magnetic flux to each moiré supercell and thus ferrimagnetism. By combining the WSe_2_/WS_2_ moiré heterobilayer with a similarly sized honeycomb superlattice (such as twisted bilayer graphene), the antiferromagnetic/ferrimagnetic patterns from shift current vortices could provide the needed staggered magnetic fields through proximity effects to generate optically controlled topological phases based on concepts such as those from the Haldane model (48).

The intrinsic properties (field intensity, shape, and orientation) of these moiré-scale magnetic nanopatterns could be efficiently controlled by the frequency, strength, and polarization of the incident light. For the linearly polarized light, along the real-space path AA-M-AA shown in Fig. 3*E*, the position for the positive and negative peaks of the induced $B_{z}$ appears alternatively with a distance about 4 nm, and the sign of the $B_{z}$ peaks may be flipped upon changing the light polarization from the *x*- to the *y*-direction (or vice versa). For the circularly polarized light, the left-handed circular polarization (LCP) and right-handed circular polarization (RCP) are related by time-reversal symmetry. As shown in Fig. 3 *C*, *D*, and *F*, upon changing the incident light from LCP to RCP (vice versa), the circulation chirality of the current vortices and the sign of induced magnetic field flip, but the magnetic field magnitude remains the same. It is worth noting that distinguished from common orbital magnetism which is at the atomic scale (with spatial extent of a few Å), the photocurrent vortices and induced magnetic nanopatterns here have the size of the moiré scale (several nm), providing much higher possibilities of being detected and manipulated in real experiments and applications. Spin-polarized scanning tunneling microscope (SP-STM) is a powerful experimental tool that can spatially resolve complex magnetic structures with an angstrom-level resolution (49–51), offering great potential to observe the real-space distributions of the predicted nanoscale magnetic patterns. Moreover, for the ferrimagnetic patterns (Fig. 3 *C* and *D*) which exhibit net finite magnetic field, other magnetic detection techniques, such as superconducting quantum interference device (SQUID) (26), could also be employed to measure the averaged field over an extensive surface area of the moiré heterobilayer.

With a moderate incident light intensity of $1.0\times 10^{10}$ W/m^2^, the maximum calculated 2D photocurrent density $J\left(r\right)$ and induced local magnetic field $B_{z}\left(r\right)$ (evaluated on the top of the Se atomic plane) for the WSe_2_/WS_2_ heterobilayer can reach 0.48 (0.13) A/m and 360 (70) nT for circularly (linearly) polarized light. As shown in Fig. 3 *G* and *H*, the strength of $J\left(r\right)$ and $B_{z}\left(r\right)$ can be tuned by the light intensity (defined as $c\varepsilon_{0}{\left|E\right|}^{2}/2$ where c and $\varepsilon_{0}$ are the light speed and permittivity of vacuum, respectively). The linear relationship between $J\left(r\right)$ or $B_{z}\left(r\right)$ with light intensity originates from the second-order nature (Eq. **2**) of the DC photocurrent response: $B_{z}\left(r\right)\propto J\left(r\right)\propto E^{2}$. With the light intensity increases to $10^{11}$ W/m^2^ in Fig. 3 *G* and *H*, the local current density rises to several A/m and the induced magnetic field is up to the order of magnitude of μT. In experiments (52), laboratory laser intensity can typically reach to $10^{13}$ W/m^2^, which is expected to give rise to an induced magnetic field as large as hundreds of μT or even higher, comparable to the Earth’s magnetic field strength (about 50 μT).

Our results thus provide a promising all-optical control route to generate and manipulate DC microscopic shift current flows in TMD moiré superlattices. For the orientation-aligned (or small angle misalignment) WSe_2_/WS_2_ moiré superlattice, setting the frequency of the incident light to excite the moiré charge-transfer excitons (peak III), a 2D shift current vortex crystal and induced magnetic field occur. We demonstrate that the location, shape, and circulation chirality as well as magnitude of the shift current vortices and hence those of the induced magnet fields can be effectively tuned by the frequency, polarization, and strength of the incident light. This is expected to be a general phenomenon for moiré superlattices with nanoscale excitons. Our results provide further understanding to nonlinear light-mater interaction in moiré quantum matter and reveals rich moiré exciton physics of shift currents.

## Methods

### Moiré Structural Relaxation and Ground-State Electronic Structure Calculations.

The structural relaxation of the rotationally aligned WSe_2_/WS_2_ moiré superlattice is performed using force fields with the LAMMPS package (53) with the help of the TWISTER code (54). The moiré supercell contains 25 × 25 WSe_2_ and 26 × 26 WS_2_ unit cells with pristine cell lattice constants of 3.32 and 3.19 Å, respectively, yielding a moiré period of about 8.3 nm. To simulate the experimental setups (32), we encapsulate the moiré bilayer by a layer of hexagonal boron nitride (*h*BN) for the structural relaxation. The Stillinger-Weber potential (55) and Kolmogorov-Crespi potential (56, 57) are used for describing the intralayer and interlayer atomic interactions for the TMD materials, respectively. In our calculations, at the reconstructed geometry, the tolerance of the force at each atom is taken as 10^−4^ eV/Å.

The ground-state electronic properties (mean-field orbital energies, wavefunctions, etc.) are obtained by density functional theory (DFT) with the Quantum Espresso package (58). We use Optimized Norm-Conserving Vanderbilt (ONCV) pseudopotential (59, 60) and an exchange-correlation functional in the generalized gradient approximation (GGA) (61) in the DFT calculations. A planewave basis with an energy cutoff of 40 Ry is used for calculating and expanding the orbital wavefunctions.

### Excited-States and Linear Optical Response Calculations.

The calculations of quasiparticle states based on the *GW* method (44) and of excitons and linear optical properties (45, 46) based on the *GW* plus Bethe–Salpeter equation (*GW*-BSE) approach are performed using the BerkeleyGW package (62). The BSE is an eigenvalue equation for two-particle exciton states:

[3] $$\left(E_{c,k}-E_{v,k}\right)A_{vc,k}^{S}+\sum_{v^{\prime }c^{\prime }{,k}^{\prime }}\langle vc,k\left|K\right|v^{'}c^{'},k^{'}\rangle A_{v^{\prime }c^{\prime }{,k}^{'}}^{S}=\Omega_{S}A_{vc,k}^{S},$$

where $E_{c,k}$ and $E_{v,k}$ are quasiparticle energies of conduction and valence states, K is the electron-hole interaction kernel, $\Omega_{S}$ is the exciton eigenvalue, and $A_{vc,k}^{S}$ is the exciton eigenvector (in basis of k-space interband transitions) with exciton index S. After solving the BSE, the real-space moiré exciton wavefunctions are expressed as

[4] $$\chi_{S}\left(r_{e},r_{h}\right)=\sum_{vc,k}A_{vc,k}^{S}\psi_{c,k}\left(r_{e}\right)\psi_{v,k}^{*}\left(r_{h}\right),$$

where $\psi_{c,k}$ ($\psi_{v,k}$) are Bloch wavefunctions of conduction (valence) bands, and $r_{e}\left(r_{h}\right)$ are electron (hole) coordinates. In Fig. 1*D*, the real-space distributions of the electron densities $\rho_{e}\left(r_{e}\right)$ and hole densities $\rho_{h}\left(r_{h}\right)$ forming the excitons are defined as $\;\rho_{e}\left(r_{e}\right)=\int {\left|\chi_{S}\left(r_{e},r_{h}\right)\right|}^{2}dr_{h}$ and $\rho_{h}\left(r_{h}\right)=\int {\left|\chi_{S}\left(r_{e},r_{h}\right)\right|}^{2}dr_{e}$ where the integrals are taken over the whole crystal. $\rho_{e}\left(r_{e}\right)$ ($\rho_{h}\left(r_{h}\right)$) corresponds to the density of the excited electron (hole) given that the hole (electron) is anywhere within the crystal.

The low-energy optical absorbance for the WSe_2_/WS_2_ bilayer with in-plane light polarization is dominated by the WSe_2_ intralayer excitons, because the interlayer excitations have negligible oscillator strength and the WS_2_ intralayer excitations have high energies which are well separated from the WSe_2_ exciton resonances (32–34). Therefore, the low-energy photoexcitation properties of the WSe_2_/WS_2_ moiré bilayers could be well approximated by those of moiré-reconstructed WSe_2_ monolayer. The accuracy of such approximation has been verified in previous studies (32, 33).

### Photoexcited Shift Current Calculations.

The photoexcited shift current coefficients and the real-space distributions of the microscopic shift current density are computed by the ab initio TD-a*GW* approach, with real-time propagation of the density matrix in the presence of the external light field (22). In this theoretical framework, the time-dependent interacting density matrix is given by

[5] $$i\text{ℏ}\frac{\partial }{dt}\rho_{nm,k}\left(t\right)={\left[H^{aGW}\left(t\right),\rho \left(t\right)\right]}_{nm,k},$$

where n and m are band indices, and $\rho_{nm,k}\left(t\right)$ is the interacting density matrix in the Bloch-state basis which is the key quantity to compute light-induced phenomena. $H_{nm,k}^{\mathrm{aGW}}\left(t\right)$ is the TD-a*GW* Hamiltonian defined as $H_{nm,k}^{\mathrm{aGW}}\left(t\right)=h_{nm,k}+U_{nm,k}^{\mathrm{ext}}\left(t\right)+\Delta V_{nm,k}^{\mathrm{ee}}\left(t\right),$ where $h_{nm,k}$ is the equilibrium quasi-particle energies which includes all the interactions at equilibrium (before application of an external optical field) at the *GW* level. The external field part is given by $U_{nm,k}^{\mathrm{ext}}\left(t\right)$ which denotes the light-matter interaction and is equal to $-eE\left(t\right)\cdot d_{nm,k}$, where E(t) is the optical electric field, and $d_{nm,k}$ is the dipole matrix (i.e., matrix element of the electron position operator ***r***) which is computed using Berry connections, with particular treatments of the intraband parts (n=m) performed with a local smooth gauge method (22). In our calculations, a dephasing factor of 10 meV is used (added to Eq. **5**) to simulate typical experimental spectral broadening (32).

Importantly, excitonic (electron-hole interaction) effects within the TD-a*GW* approach are accurately captured by the photon-field driven time variations in the electron-electron interaction term $\Delta V_{nm,k}^{\mathrm{ee}}\left(t\right)=\Delta V_{nm,k}^{H}\left(t\right)+{\Delta \Sigma }_{nm,k}^{\mathrm{COHSEX}}\left(t\right)$, where the first term is the change in the Hartree potential and the second term is the change in the electron self-energy which is taken to be the nonlocal Coulomb hole plus screened-exchange (COHSEX) *GW* self-energy in the static limit. The accuracy of the electron-hole interactions in TD-a*GW* is shown to be at the standard *GW*-BSE level, which is validated by the identical linear absorbances computed with the two methods as shown in Fig. 1*C*. Time evolution simulations excluding the electron-electron interaction $\Delta V_{nm,k}^{\mathrm{ee}}\left(t\right)$ term yield results corresponding to making the time-dependent independent-particle (TD-IP) approximation. More information on the formalism and computational details of the TD-a*GW* method can be found in ref. 22 and *SI Appendix*.

Calculating the electron-hole interaction kernel with the ab initio *GW*-BSE and TD-a*GW* methods through brute force would be computationally intractable for large-area moiré systems since there are thousands of atoms in the moiré cell. To overcome this challenge, we use the pristine unit-cell matrix projection (PUMP) method (32, 33). Employing the PUMP method, we express the moiré electronic band (quasiparticle) states as a linear combination of pristine unit-cell states, and we use the resulting expansion coefficients to rewrite the moiré electron-hole kernel matrix elements as coherent linear combinations of pristine unit-cell kernel matrix elements. The resulting ab initio BSE and TD-a*GW* calculations are performed using 12 moiré valence bands, 12 moiré conduction bands, and a 6 × 6 × 1 k-point sampling of the moiré BZ.

In calculating the shift current response at a particular frequency $\omega_{0}$, a monochromatic light field $E\left(t\right)=E_{0}\sin\left(\omega_{0}t\right)$ is employed and the photocurrent density is calculated from the expectation value of the velocity operator using the resulting time-dependent density matrix. The microscopic current density $J\left(r,t\right)$ is obtained by evaluating Eq. **1** above, once we have obtained $\rho_{nm,k}\left(t\right)$. The macroscopic current density $\bar{J}\left(t\right)$ (cell averaging of $J\left(r,t\right)$) is given by refs. 22 and 63

[6] $$\bar{J}\left(t\right)=\frac{e}{N_{k}\Omega }\sum_{nm,k}\rho_{nm,k}\left(t\right)v_{mn,k}.$$

The macroscopic shift current density can be obtained by taking its DC component ${\bar{J}}^{\mathrm{DC}}={\bar{J}}_{\omega =0}$ after performing Fourier analysis. The macroscopic shift current conductivity tensor $\sigma_{\mathrm{abc}}$ at $\omega_{0}$ are then computed from Eq. **2** above once we obtained ${\bar{J}}^{\mathrm{DC}}$.

The photocurrent-induced magnetic field is obtained from the 3D microscopic current density through the Biot–Savart law:

[7] $$B\left(r\right)=\frac{\mu_{0}}{4\pi }\int \frac{J\left(r^{\prime }\right)\times \left(r^{\prime }-r\right)}{{\left(r^{\prime }-r\right)}^{3}}dr^{\prime },$$

where $\mu_{0}$ is the permeability of vacuum. The integral is performed over the whole space. In Fig. 3, the $B\left(r\right)$ is evaluated on the top of Se atomic plane for the WSe_2_ layer.

## Supplementary Material

Appendix 01 (PDF)

## Data Availability

All study data are included in the article and/or *SI Appendix*.
