# Supplementary material for: Light-induced shift current vortex crystals in moiré heterobilayers
Source: Proc Natl Acad Sci U S A. 2023 Dec 12;120(51):e2314775120. doi: 10.1073/pnas.2314775120 (PMC10741382; doi:10.1073/pnas.2314775120)
Supplement: Supplementary file 1 — Appendix 01 (PDF) [file pnas.2314775120.sapp.pdf]

PNAS

Supporting Information for

Light-induced shift current vortex crystals in moiré heterobilayers

Chen Hu[1,2], Mit H. Naik[1,2], Yang-Hao Chan[1,2,3], Jiawei Ruan[1,2], and Steven G. Louie[1,2,*]

[1] Department of Physics, University of California at Berkeley, Berkeley, California 94720, USA

[2] Materials Sciences Division, Lawrence Berkeley National Laboratory, Berkeley, California 94720, USA

[3] Institute of Atomic and Molecular Sciences, Academia Sinica, and Physics Division, National Center for Theoretical Sciences, Taiwan

*Email: sglouie@berkeley.edu

In this Supporting Information, we provide some detailed computational information and further results. We organize the material into the following sections:

1. Computational details of GW and *GW*-BSE calculations.
2. Formalism and details of TD-a*GW* calculations.
3. Dependence of shift current vortices on polarization direction of linearly polarized light.

1. Computational details of *GW* and *GW*-BSE calculations

In our study, we compute the quasiparticle self-energy corrections to the Kohn-Sham orbital energies of pristine WSe_2 at the G_0W_0 level[1], and the same corrections to the band states including spin-orbital effects are used in the reconstructed WSe_2. During the *GW* calculations, 6000 bands and a 30 Ry cutoff are included. The nonuniform neck subsampling (NNS) method[2] is used to describe the 2D dielectric screening. Dynamical screening is treated by using the Hybertsen-Louie generalized plasmon pole model[1]. For the *GW*-BSE calculations on excitons and optical absorbance[3,4], a $6 \times 6 \times 1$ k-grid of the moiré Brillouin zone (BZ) is employed (equivalent to a k-point sampling of $150 \times 150 \times 1$ in the pristine BZ). 12 valence bands and 12 conduction bands of the moiré-reconstructed WSe_2 are used.

2. Formalism and details of TD-aGW calculations

For interacting quantum many-body systems, the Kadanoff-Baym equations[5] (KBE) approach provides a general theoretical framework to study nonequilibrium physical phenomena, which describes the equations of motion for the interacting Green's function G on the Keldysh contour C:

$$i\frac{d}{dt}G(t,t') = \delta(t,t') + [H_0 + U^{ext}(t)]G(t,t') + \int_C \Sigma(t,t'')G(t'',t')dt'' \ , \qquad (S1)$$

where G is the contour-ordered Green's function, H_0 is the equilibrium Hamiltonian, $U^{ext}(t)$ is the interaction with an external field, and Σ denotes the electron self-energy. Besides Eq. S1, the full set of KBE also includes another adjoint equation which propagates in t'. The coupled propagations of the two time variables (t and t') bring in tremendous computational burden for directly solving KBE. Here we adopted a practical and effective method called the adiabatic GW (aGW) approximation[6,7], in which an instantaneous self-energy (time-diagonal $t = t'$), *i.e.,* a static Coulomb hole plus screened-exchange (COHSEX) self-energy is employed.

Within the framework of TD-aGW approach, the KBE can be rigorously expressed as an equation of motion of the density matrix (Eq. 5 of the main text). In our calculations, we utilize a Bloch-state basis for operators: $O_{nm,\boldsymbol{k}} = \langle n\boldsymbol{k}|\hat{O}|m\boldsymbol{k}\rangle$, where $|n\boldsymbol{k}\rangle$ and $|m\boldsymbol{k}\rangle$ are Bloch eigenstates, and $\hat{O}$ is the operator of a physical quantity of interest, such as the Hamiltonian H, the density matrix ρ, self-energy Σ, *etc*.

In the TD-aGW approach, electron-hole interactions (excitonic effects) are included through the change in the self-energy $\Delta\Sigma_{nm,\boldsymbol{k}}^{COHSEX}(t)$ given as[6]:

$$\Delta\Sigma_{nm,\boldsymbol{k}}^{COHSEX}(t) = i\sum_{n'm',\boldsymbol{k}'} \Delta G_{n'm',\boldsymbol{k}'}^{<}(t)\ W_{n\boldsymbol{k},n'\boldsymbol{k}';\,m\boldsymbol{k},m'\boldsymbol{k}'}\ , \qquad (S2)$$

where $G_{n'm',\boldsymbol{k}'}^{<}(t)$ is the time-diagonal of the lesser Green's function and we have $\rho_{nm,\boldsymbol{k}}(t) = -iG_{nm,\boldsymbol{k}}^{<}(t)$. W is the statically screened Coulomb interaction, as used in the standard formulation of the electron-hole interaction kernel of the GW-BSE Hamiltonian:

$$W_{n\boldsymbol{k},n'\boldsymbol{k}';\,m\boldsymbol{k},m'\boldsymbol{k}'} = \iint \psi_{n\boldsymbol{k}}^{*}(\boldsymbol{r})\psi_{n'\boldsymbol{k}'}(\boldsymbol{r})W(\boldsymbol{r},\boldsymbol{r}')\,\psi_{m\boldsymbol{k}}(\boldsymbol{r}')\psi_{m'\boldsymbol{k}'}^{*}(\boldsymbol{r}')\,d\boldsymbol{r}\,d\boldsymbol{r}'. \qquad (S3)$$

It is proved that by employing $\Delta\Sigma_{nm,\boldsymbol{k}}^{COHSEX}(t)$ within the TD-aGW approach, one can fully capture the electron-hole interactions in linear optical processes at the GW-BSE level[6,7]. This is evidenced

by the identical linear absorbance from the TD-aGW method and GW-BSE method shown in Fig. 1c of the main text. The TD-aGW approach goes beyond standard GW-BSE method in that it yields nonlinear and time-dependent optical responses including electron-hole interactions.

In our TD-aGW calculations, the time propagation is performed with a time step of 0.0048 fs using a fourth order Runge-Kutta method. We employ 12 valence bands, 12 conduction bands, a $6 \times 6 \times 1$ k-grid of the moiré BZ (equivalent to $150 \times 150 \times 1$ in pristine BZ) for the TD-aGW simulations, consistent with our GW-BSE calculations.

3. Dependence of shift current vortices on polarization direction of linearly polarized light

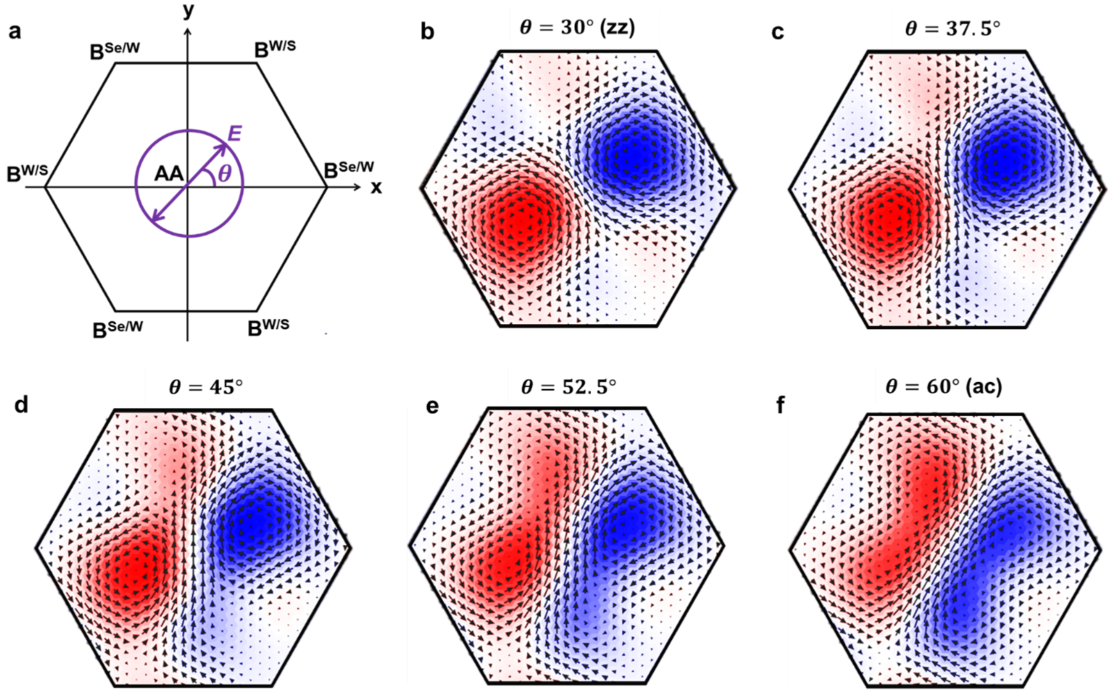

Fig. S1: Real-space plots of the microscopic shift current density (arrows) and the current-induced magnetic field (color coding) for light with frequency of that of exciton peak III (1.87 eV) and with different linear light polarization directions.

In Fig. 3a,b of the main text, we have presented plots of the microscopic shift current density with linear light polarizations along the x-direction ($\theta = 0°$) and the y-direction ($\theta = 90°$), corresponding to the armchair (ac) and zigzag (zz) direction, respectively. Due to the C_{3v} symmetry of the moiré superlattice, linear light polarization along other armchair or zigzag direction will induce identical current density but rotated accordingly (comparing Fig. S2f with Fig. 3a or comparing Fig. S2b with Fig. 3b). Changing the linear light polarization direction from $\theta = 30°$

to 60°, as shown in Fig. S2b-f, one can tune the shift current vortices and the induced magnetic field distributions continuously.

References of SI:

1. M. S. Hybertsen, S. G. Louie, Electron correlation in semiconductors and insulators: band gaps and quasiparticle energies. Phys. Rev. B. 34, 5390-5413 (1986)
2. F. H. da Jornada, D. Y. Qiu, S. G. Louie, Nonuniform sampling schemes of the Brillouin zone for many-electron perturbation-theory calculations in reduced dimensionality. Phys. Rev. B 95, 035109 (2017)
3. M. Rohlfing, S. G. Louie, Electron-hole excitations in semiconductors and insulators, Phys. Rev. Lett. 81, 2312 (1998)
4. M. Rohlfing, S. G. Louie, Electron-hole excitations and optical spectra from first principles. Phys. Rev. B. 62, 4927-4944 (2000)
5. L. P. Kadanoff, G. Baym, Quantum Statistical Mechanics (W. A. Benjamin, Inc., 1962)
6. Y. -H. Chan, D. Y. Qiu, F. H. da Jornada, S. G. Louie, Giant exciton-enhanced shift currents and direct current conduction with subbandgap photo excitations produced by many-electron interactions. Proc. Natl. Acad. Sci. U.S.A. 118, e1906938118 (2021)
7. C. Attaccalite, M. Grüning, A. Marini, Real-time approach to the optical properties of solids and nanostructures: Time-dependent Bethe-Salpeter equation. Phys. Rev. B 84, 245110 (2011)
